# Complete Genome Sequence of Pasteurella multocida HuN001, a Capsular Type A Strain from a Human

**DOI:** 10.1128/MRA.00395-21

**Published:** 2021-07-01

**Authors:** Lin Lin, Chunhui Li, Fei Wang, Xueying Wang, Yue Zhang, Songtao Liu, Wan Liang, Lin Hua, Zhong Peng, Bin Wu

**Affiliations:** a State Key Laboratory of Agricultural Microbiology, College of Veterinary Medicine, Huazhong Agricultural University, Wuhan, China; b Infection Control Center, Xiangya Hospital of Central South University, Changsha, China; c Ministry of Science and Technology International Research Center for Animal Disease, The Cooperative Innovation Center for Sustainable Pig Production, Huazhong Agricultural University, Wuhan, China; d Ministry of Agriculture and Rural Affairs Key Laboratory of Prevention and Control Agents for Animal Bacteriosis, Institute of Animal Husbandry and Veterinary Sciences, Hubei Academy of Agricultural Sciences, Wuhan, China; University of Arizona

## Abstract

Here, we report the complete genome sequence of clinical Pasteurella multocida strain HuN001, which was cultured from a sputum sample from a patient with pneumonia. Oxford Nanopore Technologies sequencing provided a complete genome sequence of P. multocida HuN001, which contains a 2,287,216-bp circular chromosome with an average G+C content of 40.33%.

## ANNOUNCEMENT

Pasteurella multocida is an important zoonotic pathogen that mainly causes respiratory symptoms as well as hemorrhagic septicemia in multiple animal species and even in humans (1, 2). P. multocida strains recovered from different host species are classified into five capsular serogroups (A, B, D, E, and F) (3) or genotypes (A, B, D, E, and F) (4) and 16 lipopolysaccharide (LPS) serovars (serovars 1 to 16) (5) or eight LPS genotypes (L1 to L8) (6). Recently, the public availability of increasing numbers of P. multocida genome sequences has facilitated great progress in increasing the knowledge of both bacterial typing and the pathogenesis of P. multocida (1). However, most of these publicly available genome sequences are those of P. multocida strains that originated from animals, and very few of them are from P. multocida strains of human origin. Here, we describe the complete genome analysis of a P. multocida strain that was isolated from a pulmonary infection.

**FIG 1:**
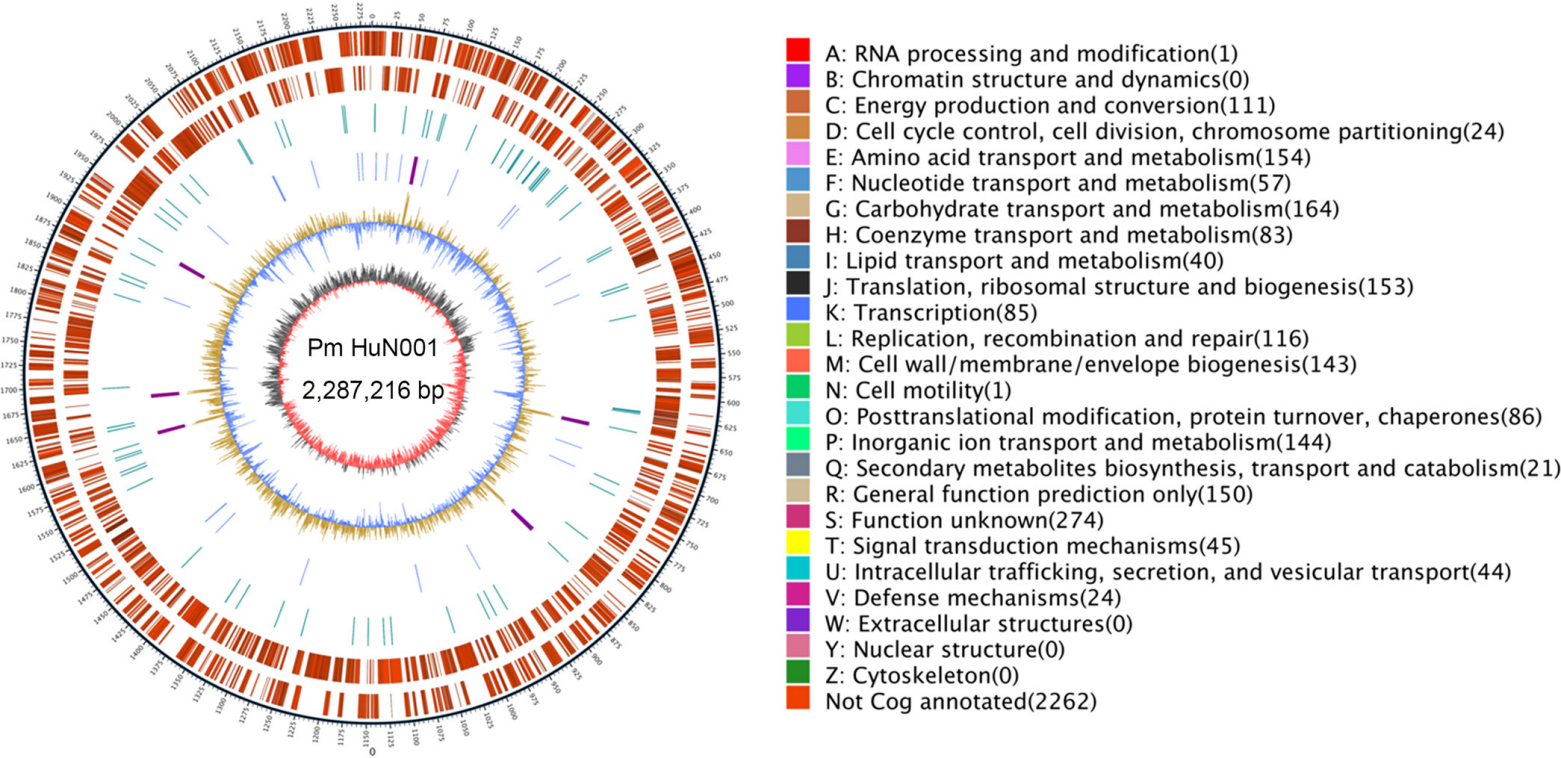
Genomic characteristics of the complete genome sequence of P. multocida HuN001. (Left) Circular map of the complete genome sequence. From inside to outside, the circles represent GC skew (circle 1), G+C content (circle 2), positions of tRNAs (in blue) and rRNAs (in purple) (circle 3), repeat sequences (circle 4), genes located on the negative strand (circle 5), and genes located on the positive strand (circle 6). (Right) Clusters of Orthologous Genes (COG) functions of different genes.

P. multocida strain HuN001 was isolated from a sputum sample from a patient with pneumonia in Hunan Province, China. Briefly, the sputum sample from the patient was streaked onto tryptic soy agar (TSA) (Becton, Dickinson and Co., Sparks, MD, USA) supplemented with 5% newborn calf serum (Tianhang Biotechnology, Hangzhou, China), which was incubated overnight at 37°C. After that, single colonies were picked for Gram staining and 16S rRNA gene sequencing to determine the genus and species of the bacterial isolates. This process is a routine procedure for diagnosis and has been approved by the Ethics Committee of Xiangya Hospital (Changsha, China). Genomic DNA of the isolate was extracted from a bacterial culture of a single colony at 37°C in tryptic soy broth (TSB) (Becton, Dickinson and Co.) supplemented with 5% bovine serum using the QIAamp DNA minikit (Qiagen, Hilden, Germany). DNA quality and quantity were assessed using electrophoresis on a 0.35% agarose gel and were double checked by NanoDrop spectrophotometry (Thermo Fisher Scientific, USA) and Qubit 3.0 fluorometry (Thermo Fisher Scientific), respectively. Then, 20-kb to 30-kb DNA libraries were generated using an SQK-LSK109 ligation sequencing kit (Oxford Nanopore Technologies [ONT], Oxford, UK) and were sequenced on a PromethION platform (ONT), according to the manufacturer’s protocol, at BioMarker Technologies Corp. (Beijing, China). Base calling was performed using the Nanocall package (https://github.com/mateidavid/nanocall) with default parameters (7). The strategy yielded 1,582,081,118 bp of raw reads (*N*_50_, 16,755 bp; *N*_90_, 8,425 bp), and a total of 1,448,813,812 bp of filtered data (*N*_50_, 16,900 bp; *N*_90_, 8,815 bp) was finally obtained after removal of reads with mean_qscore_template of <7 and length of <2,000 bp. The Canu v1.5 (8) package was then used to assemble the filtered data, and the quality of the assembly was double checked using Racon v3.4.3 software and Circlator v1.5.5 software with default parameters. Finally, a single circular chromosome 2,287,216 bp in length was generated for the complete genome sequence of P. multocida strain HuN001 (Fig. 1 and Table 1).

**TABLE 1:** General features of the complete genome sequence of P. multocida HuN001

Genomic feature	Value
Total length (bp)	2,287,216
G+C content (%)	40.33
Contig *N*_90_ (bp)	2,287,216
Contig *N*_50_ (bp)	2,287,216
Avg coverage (×)	691.7
No. of genes	2,127
Total repetitive sequence length (bp)	3,747
No. of rRNAs	19
No. of 16S rRNAs	6
No. of 23S rRNAs	6
No. of 5S rRNAs	7
No. of tRNAs	58
No. of genomic islands	3
No. of prophages	2
No. of virulence factors (VFDB)	353
No. of resistance factors (CARD database)	3
No. of secreted proteins	197

The public version of the genome was annotated by the NCBI Prokaryotic Genome Annotation Pipeline (PGAP) (9); through this strategy, we found that the complete genome of P. multocida HuN001 contained 2,127 protein-coding genes, 19 rRNAs, and 58 tRNAs. In particular, a number of genes associated with bacterial adherence and invasion, capsule and LPS biosynthesis, iron acquisition and uptake, sialic acid metabolism, and outer membrane formation were identified in the chromosome using the Virulence Factor Database (VFDB) (10). However, only three antimicrobial resistance determinants, for β-lactams (GE000922) and pulvomycin (GE001297 and GE001710), were identified through the CARD database (11). Genotyping using the PmGT online tool (http://vetinfo.hzau.edu.cn/PmGT/) revealed that P. multocida HuN001 was assigned as capsular, LPS genotype A, and L1. Strikingly, it was assigned as a novel sequence type because no sequence type was given by the online tool, even though the allele numbers of each of the housekeeping genes used for multilocus sequence typing (*adk*, *aroA*, *deoD*, *gdhA*, *g6pd*, *mdh*, and *pgi*) were obtained.

### Data availability.

The complete genome sequence of P. multocida strain HuN001 has been deposited in GenBank under accession number CP073238. The raw reads are available in the NCBI Sequence Read Archive (SRA) under accession number SRR14253068. The BioProject accession number is PRJNA722379, and the BioSample accession number is SAMN18753491.
